# Circulating Bacterial DNA in Colorectal Cancer Patients: The Potential Role of *Fusobacterium nucleatum*

**DOI:** 10.3390/ijms25169025

**Published:** 2024-08-20

**Authors:** Ioannis Koliarakis, Ilias Lagkouvardos, Konstantinos Vogiatzoglou, Ioannis Tsamandouras, Evangelia Intze, Ippokratis Messaritakis, John Souglakos, John Tsiaoussis

**Affiliations:** 1 Department of Anatomy, School of Medicine, University of Crete, 70013 Heraklion, Greece; medp2011931@med.uoc.gr; 2 Department of Clinical Microbiology, School of Medicine, University of Crete, 70013 Heraklion, Greece; ilias.lagkouvardos@hcmr.gr (I.L.); medp2012153@med.uoc.gr (E.I.); 3 Laboratory of Translational Oncology, Medical School, University of Crete, 70013 Heraklion, Greece; medp2012081@med.uoc.gr (K.V.); messaritakis@uoc.gr (I.M.); sougl@uoc.gr (J.S.); 4 Department of Otorhinolaryngology—Head and Neck Surgery, University General Hospital of Heraklion, 71110 Heraklion, Greece; ioantsam3@med.duth.gr; 5 Department of Microbiology, German Oncology Center, Yiannoukas Labs LTD, Bioiatriki Group, Limassol 4108, Cyprus; 6 Department of Medical Oncology, University Hospital of Heraklion, 71110 Heraklion, Greece

**Keywords:** colorectal cancer, dysbiosis, bacterial translocation, surgery, intestinal microbiota, circulating bacterial DNA, metastasis, *Fusobacterium nucleatum*

## Abstract

Intestinal dysbiosis is a major contributor to colorectal cancer (CRC) development, leading to bacterial translocation into the bloodstream. This study aimed to evaluate the presence of circulated bacterial DNA (cbDNA) in CRC patients (*n* = 75) and healthy individuals (*n* = 25). DNA extracted from peripheral blood was analyzed using PCR, with specific primers targeting *16S* rRNA, *Escherichia coli* (*E. coli*), and *Fusobacterium nucleatum* (*F. nucleatum*). High *16S* rRNA and *E. coli* detections were observed in all patients and controls. Only the detection of *F. nucleatum* was significantly higher in metastatic non-excised CRC, compared to controls (*p* < 0.001), non-metastatic excised CRC (*p* = 0.023), and metastatic excised CRC (*p* = 0.023). This effect was mainly attributed to the presence of the primary tumor (*p* = 0.006) but not the presence of distant metastases (*p* = 0.217). The association of cbDNA with other clinical parameters or co-morbidities was also evaluated, revealing a higher detection of *E. coli* in CRC patients with diabetes (*p* = 0.004). These results highlighted the importance of bacterial translocation in CRC patients and the potential role of *F. nucleatum* as an intratumoral oncomicrobe in CRC.

## 1. Introduction

Colorectal cancer (CRC) is the third most common cancer worldwide and a leading cause of cancer mortality, accounting for 9% of all malignancies in adult patients [1]. Gradual accumulation of genetic mutations results in the formation of pre-cancerous lesions, which subsequently evolve into colorectal adenoma and, ultimately, invasive CRC [2]. Immune and inflammatory responses are crucial in all stages of colorectal tumorigenesis [3]. Earlier CRC detection has been made feasible through current innovations in screening endoscopy, imaging modalities, and therapeutic strategies (surgery, radiotherapy, and chemotherapy), leading to enhanced survival rates [4].

Up to 22% of CRC patients are identified with metastatic disease at diagnosis, and various studies indicate that approximately 70% of patients inevitably exhibit metastatic disease or recurrence, with up to 50% of cases involving synchronous or metachronous distant metastases [5]. Stage IV CRC patients typically have a poor prognosis, with a 14% 5-year survival rate. Hence, the development of efficient prognostic or diagnostic biomarkers is still a necessity.

The correlation between CRC and intestinal microbiota has been extensively evidenced. The diverse microbial ecosystem of the human intestinal microbiota constantly interacts with the host, maintaining homeostasis through constant synergistic interactions with the host. However, disruptions in microbiota composition could favor the proliferation of pathogenic bacteria, leading to detrimental effects, collectively termed “dysbiosis” [6]. Apart from alterations in microbial composition, dysbiosis also encompasses shifts in bacterial distribution and dysregulated metabolism, promoting colorectal tumorigenesis [7]. Such effects could lead to DNA damage, modulation of immunity, and an inflammatory response [8]. Interestingly, surgical stress subsequent to curative resection for CRC affects the host–microbiota interaction, further promoting dysbiosis [9]. Alterations in various bacterial genera, including *Bacteroides*, *Bifidobacterium*, *Escherichia*, *Fusobacterium*, and *Lactobacillus*, among others, have been demonstrated after CRC surgery [10]. *Fusobacterium nucleatum* (*F. nucleatum*), an invasive, pro-inflammatory pathogen, indigenous to the oral microbiota [11], is one of the most researched bacterial species in CRC. *F. nucleatum* triggers carcinogenesis mainly by stimulating the *β*-catenin cascade following the binding of the Fusobacterium adhesion A (FadA) protein to E-cadherin [12]. Various other mechanisms of colorectal tumorigenesis mediated by *F. nucleatum* include the upregulation of microRNA-21 through the stimulation of toll-like receptor (TLR) 4, the induction of Th17 responses through the increased production of interleukin (IL)-23, and causing DNA damage via the modulation of base excision repair (BER) [12]. Studies associate the higher abundance of *F. nucleatum* in metastatic disease with the reduced overall or cancer-specific survival in CRC patients [13].

Circulating bacterial DNA (cbDNA) in human blood has become evident [14]. Detection of cbDNA, through various molecular techniques [15,16], has also been reported as a reliable, non-invasive method for CRC screening and prediction of long-term outcomes in CRC patients, possibly participating in CRC pathogenesis [17,18]. However, there are limited data regarding the detection of cbDNA in patients with metastatic CRC, especially concerning any differences regarding the absence of the primary tumor due to previous surgical resection, and focusing on the presence of *F. nucleatum*. We hypothesize that the presence of the primary tumor with distant metastases could be associated with alterations in the cbDNA of CRC patients.

In this context, the present study aimed to investigate the detection of cbDNA in the blood of patients with CRC using a polymerase chain reaction (PCR)-based method, to compare patients with or without surgical resection of the primary tumor, as well as non-metastatic and metastatic diseases, and to evaluate any associations with the patients’ demographic and clinical parameters.

## 2. Results

### 2.1. Patients’ Characteristics

The epidemiological and clinical characteristics of patients and healthy individuals are summarized in Table 1 and Data S1. Patient groups with CRC were older (mean age range 57.8–62.9 years old) and had a higher body mass index (BMI) (mean BMI range 27.7–28.4 kg/m^2^), compared to Group 1. Most of the total participants (63%) were males. By design, all patients in Groups 2 and 3 underwent surgical resection of the primary tumor, whereas all patients in Groups 3 and 4 had metastatic disease (stage IV CRC). The majority of Group 2 patients (80%) had locally advanced (stage III) CRC. In Group 4 patients, the tumor location was evenly distributed between the left colon and the rectum (40% each), whereas the most common CRC site regarding non-metastatic groups was the right colon (40% in Groups 2 and 3, respectively). Pathologic staging of CRC was mostly intermediate (58.7% of all patients), while tumors presented microsatellite instability (MSI) in only 9.3% of CRC patients. The majority of patients were non-active smokers (64%) and reported no or infrequent alcohol consumption (89.3%), as determined by a negative Alcohol Use Disorders Identification Test-Concise (AUDIT-C) score (<3 in females and <4 in males). Regarding co-morbidities, most of the patients had cardiovascular disease (CVD) (45.3%), whereas diabetes and dyslipidemia were more common in Group 3 (44%) and Group 2 (36%), respectively. None of the Group 1 individuals had any co-morbidities.

### 2.2. Detection of cbDNA

**Figure 1:**
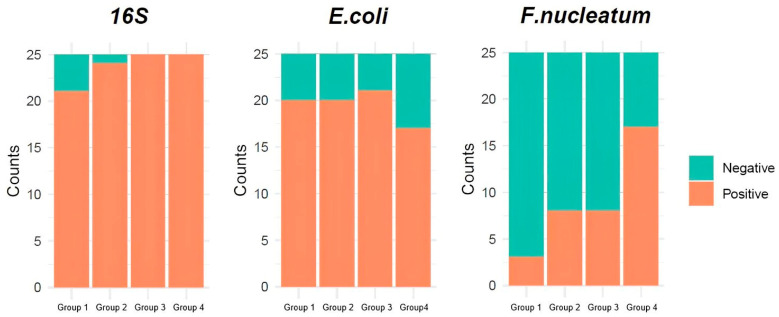
Frequencies of *16S* rRNA, *Escherichia coli* (*E. coli*), and *Fusobacterium nucleatum* (*F. nucleatum*) detection in blood among control and patient groups.

In the results, *16S* rRNA was detected in 84% of Group 1, 96% of Group 2, and 100% of Groups 3 and 4. The *β*-galactosidase gene of *Escherichia coli* (*E. coli*) was detected in 80% of Groups 1 and 2, 84% of Group 3, and 68% of Group 4. Since *F. nucleatum* has been extensively associated with metastatic CRC, having been characterized as an oncomicrobe mainly affecting the intratumoral microenvironment [13], we chose to specifically focus on its detection in the blood, since the majority of our patients’ cohorts had metastatic CRC, filling the knowledge gap in the current literature. Notably, the *NusG* gene of *F. nucleatum* was detected in 12% of Group 1, 32% of Groups 2 and 3, and 68% in Group 4 (Table 2 and Figure 1). Examples of *E. coli* and *F. nucleatum* detection are presented in Figure S1.

Pairwise comparisons between groups revealed a significantly higher detection of *F. nucleatum* in Group 4, compared to Group 1 (*p* < 0.001, adj. *p* < 0.001), as well as compared to Group 2 or 3 (*p* = 0.023, adj. *p* = 0.045). However, there were no statistically significant differences regarding the detection of *16S* rRNA or *E. coli* among all groups (Table 3).

A further correlation of *E. coli* and *F. nucleatum* presences was performed after grouping the patients according to their clinical parameters. The detection of *E. coli* and *F. nucleatum* in blood was not significantly associated with differences in sex, metastasis, tumor location, grade, mismatch repair (MMR) status, smoking, alcohol consumption, dyslipidemia, or CVD (Table 4). Patients without surgical resection of the primary tumor presented a significantly higher detection of *F. nucleatum,* compared to those with surgical resection of the primary tumor (68% vs. 32%, *p* = 0.006). However, no such difference was observed concerning the detection of *E. coli* (68% vs. 82%, *p* = 0.242). Additionally, the detection of *E. coli* was significantly higher in patients with diabetes, compared to those without (100% vs. 69.6%, *p* = 0.004), whereas such an association was not evident with the presence of *F. nucleatum* (31.6% vs. 48.2%, *p* = 0.286).

## 3. Discussion

Lately, research groups have become increasingly interested in peripheral blood as a novel valuable source of cbDNA. PCR-based methods were developed in earlier studies, enabling the detection of cbDNA in CRC patients [19,20]. However, small cohorts were enrolled, and there was no discrimination between CRC patients’ characteristics or proper integration of control subjects. More recent studies relying on advanced PCR-based methods [17,18,21,22] or next-generation sequencing (NGS) methods [23,24] shed light on the elusive subject of cbDNA detection in CRC patients.

In brief, the present study reveals that cbDNA of *16S* rRNA, *E. coli*, and *F. nucleatum* is present in the blood of healthy subjects and CRC patients. However, the origin of the cbDNA remains elusive. The presence of microbes in the blood may be attributed to occasional dissemination from various body reservoirs into the circulation, known as microbial translocation [25]. The main proposed mechanisms for this phenomenon are intestinal dysbiosis, dysfunction of the intestinal epithelial barrier, and increased permeability (“leaky-gut”). Notably, microbial components, including endotoxins, lipopeptides, and nucleic acids, among others, could also be present in the blood [26]. Additional mechanisms for bacterial translocation into the blood include the interaction of the microbiota with immune system cells, affecting multiple host functions [27], promoting chronic local and systemic inflammations [28], and utilizing dendritic cells or micro-fold cells [29]. Studies have shown that cbDNA is predominantly related to intestinal dysbiosis, although oral or skin microbiota could also serve as potential sources of cbDNA [23]. Studies have revealed great similarity of the *F. nucleatum* subtypes between saliva and tumor tissue samples in CRC [30], enhancing the hypothesis of orally-mediated intestinal dysbiosis. It is evident that in periodontitis, several oral pathogens, including *F. nucleatum*, are incorporated into complex oral biofilms, facilitating the translocation of oral pathogens into the intestinal ecosystem by invading the bloodstream or through swallowing saliva [31]. Residing in the colonic microbiota, they further promote dysbiosis. In our cohort, it is reasonable to suggest that the majority of the cbDNA could probably have originated from the intestinal or oral microbial community. Nevertheless, the primary factor, between the inflammatory response, alterations in microbiota composition, or increased intestinal permeability, leading to bacterial translocation remains unknown [32,33].

Our study demonstrated a high detection of *16S* rRNA (84%) and *E. coli* (80%) in the blood of healthy subjects, which was non-significantly different, compared to CRC patients (96–100% and 68–84%, respectively). Similar to our study, Giacconi et al. [21], by using real-time qPCR, identified the presence of *16S* rRNA in all 40 control subjects and 50 CRC patients, although the bacterial load was higher in CRC patients, compared to healthy subjects. Mutignani et al. [24], using NGS, detected *16S* rRNA in all healthy controls and subjects with colorectal adenomas; CRC patients, again, presented with a higher cbDNA load. The abundance of *E. coli* did not differentiate between non-CRC and CRC subjects. Another study by Xiao et al. [23], also using NGS, analyzed the cbDNA between healthy controls and patients with colorectal adenoma or CRC. A prominent and distinctive circulating cbDNA profile was identified between CRC patients and healthy subjects, highlighting 28 species deriving from intestinal or oral microbiota, which did not include *E. coli*. Messaritakis et al. [17], however, reported a higher PCR detection of *16S* rRNA in a larger cohort of 397 CRC patients (64.5%), compared to 32 healthy controls (15.6%). Notably, although there was no association concerning *E. coli* detection between these groups (*p* = 0.186), in accordance with our results, the percentages of positivity were significantly lower, compared to our study (15.6% in the control group and 26.2% in the CRC group).

The biological causes underlying these discrepancies are largely unknown. Concerning *16S* rRNA, our study demonstrates a potentially substantial impairment of the intestinal permeability in healthy individuals, leading to bacterial circulation, in accordance with previous reports [34]. Moreover, *E. coli* is an almost exclusively nonpathogenic commensal species of the intestinal microbiota, having been detected as a member of the intestinal microbiome in over 90% of healthy individuals [35]. The current literature regarding *E. coli* and CRC is largely ambiguous regarding its over- or under-representation in CRC-related microbiota, compared to controls. This phenomenon is possibly due to the different abundances of the various phylotypes of *E. coli* (A, B1, B2, and D), where *E. coli* strains belonging to phylotype A are mostly commensal, while strains of the B2 phylotype are mainly considered virulent species [36]. It is difficult to explain the lack of difference in *16S* rRNA or *E. coli* between CRC patients and healthy subjects, since inflammatory responses in the CRC microenvironment could also affect bacterial dissemination [37]. This outcome could be partially attributed to a variety in size or shape inclinations in the gut–blood bacterial translocation. It should further be emphasized that the present study compared healthy controls with stage IV CRC with or without surgical resection of the primary tumor and stage III CRC with surgical resection of the primary tumor, whereas in the aforementioned studies, the pool of CRC patients included patients with intestinal adenomas or stage I-III CRC without surgical resection of the primary tumor. Hence, our results are not directly comparable to these studies and should be carefully interpreted. The detection of bacterial by-products (metabolites or toxins) in the bloodstream may further aid the differentiation between tumor-free individuals and CRC patients. Future multi-omic studies integrating the analysis of cbDNA with microbiota profiling and the metabolome could unveil the molecular mechanism of cbDNA alteration in CRC tumorigenesis.

In this study, we also report a significantly higher detection of *F. nucleatum* cbDNA in CRC patients (32–68%), compared to healthy subjects (12%). This is in accordance with the higher identification of *F. nucleatum* in mucosal and fecal samples from CRC patients, promoting intestinal dysbiosis [38,39,40]. To date, only the study by Xiao et al. [23] compared the *F. nucleatum* in the blood but found no significant difference between CRC patients and the controls. However, this difference may be due to the relatively small sample size of their study, in addition to the inclusion of only earlier-stage CRC (II/III) and the different demographic or environmental variables.

Interestingly, in our setting, the detection of *F. nucleatum* cbDNA was significantly higher in the stage IV CRC patients without surgical resection of the primary tumor, compared to stage II/III CRC or stage IV CRC patients with surgical resection of the primary tumor. This finding is in line with the current evidence that *F. nucleatum* constitutes a predominantly intratumoral oncomicrobe affecting the tumor microenvironment in promoting CRC pathogenesis [41], and its presence has also been extensively correlated with advanced CRC stages [42]. Nevertheless, it has been demonstrated that the surgical resection of the primary tumor in CRC patients could enhance or reduce the abundance of *F. nucleatum* in the gut [43,44,45]. Thus, our results indicate that the resection of the primary tumor could disrupt the active oral–gut axis in stage IV CRC, reducing the circulation of *F. nucleatum*.

*E. coli* cbDNA was not significantly different between CRC patients with or without surgical resection of the primary tumor in our study. Only one study by Koulouridi et al. [18] investigated the detection of *E. coli* in the blood of stage III CRC patients with surgical resection of the primary tumor, reporting a lower frequency (21.5%) in comparison with our results (82%). Studies on the gut microbiota of CRC patients revealed reduced populations of *E. coli* in cancerous tissue, compared to adjacent healthy mucosa [46], while others reported inconsistent alterations of *E. coli* abundance in surgically treated CRC patients [45,47,48], which could merely explain these results. Research has revealed that in CRC, *E. coli* could invade the weakened intestinal vascular barrier and be released into the portal circulation, colonizing the liver and promoting liver metastasis [49]. This fact, in combination with the observation that *E. coli* is not a strictly intratumoral microbe [47,50], could explain the similarity in the detections of *E. coli* cbDNA between stage IV CRC patients with or without surgical resection of the primary tumor. Notably, we further observed that neither *E. coli* nor *F. nucleatum* cbDNA was significantly altered between non-metastatic (stage II/III) and metastatic (stage IV) CRC patients. *F. nucleatum* was thoroughly correlated with increased tumor invasion and lymph node or distant metastases [13,42], promoting metastasis by regulating signaling molecules, including IL-8, C-X-C motif chemokine ligand 1 (CXCL1), and keratin 7 (KRT7) [50]. *E. coli* has also been implicated in metastatic colorectal disease in combination with circulating tumor cells [51]. Similar to our results, Giacconi et al. [21] revealed no correlation between increased cbDNA levels and the tumor stage or the presence of distant metastases. The authors hypothesized that this finding might support the concept that cbDNA primarily plays a role in the early development of CRC. The study by Messaritakis et al. [17] reported a significantly higher presence of *E. coli* in stage IV CRC, compared to stage II/III; however, cases with or without surgical resection of the primary tumor were pooled together, which did not allow an accurate comparison with our data. Our study did not include stage II/III CRC cases without surgical resection of the primary tumor, thus presenting a further limitation in elucidating the aforementioned discrepancies regarding the cbDNA association with the CRC stage or metastases.

Tumor location was not associated with cbDNA detection in our setting, similar to previous PCR-based reports [17]. The utilization of NGS by Mutignani et al. [24] displayed an enhanced bacterial transition from the intestinal environment to blood circulation in right colon cancer, compared to rectal cancer. Regional discrepancies in genetic expression and immunological characteristics were also emphasized [52]. Furthermore, intestinal microbiota composition-related tumorigenic mechanisms also varied between the left colon, right colon, or rectum [53]. These data indicate the potential usefulness of sensitive NGS-based methods in discerning bacterial translocation according to the CRC site.

Regarding other demographic or clinical parameters of CRC patients, our study is unique in investigating any possible associations with cbDNA. Gut microbiota studies reveal a distinct microbiota profile in correlation with deficient mismatch repair [54] or high-graded CRC [55]. However, cbDNA detection in our cohorts revealed no significant differences in these characteristics. Several risk factors can contribute to intestinal dysbiosis, such as alcohol consumption [56] and smoking, as well as obesity [57]. Studies have also shown the definite presence of cbDNA in physiological conditions and various systematic diseases, including diabetes and metabolic or cardiovascular diseases [29,58]. The CRC patients in this study displayed several clinical characteristics, together with the aforementioned diseases linked to dysbiosis. Between all these factors, our study only revealed a strong correlation between the higher *E. coli* cbDNA presence in CRC patients and diabetes. The abundance of *E. coli* in the gut microbiota of patients with diabetes increased, serving as an opportunistic pathogen [59]. Nevertheless, the association of cbDNA detection with all of the above clinical factors or co-morbidities may be influenced by the impact of the small sample size involved in our research.

Apart from the aforementioned limitations, it should be noted that our study, by design, was a non-randomized prospective study. We were not able to adjust for age between CRC and the controls, with the latter belonging to a significantly lower age range, removing potential factors that could influence the results of cbDNA by controlling for these covariates. However, to mitigate the effect of the small sample size, the gender and geographical distribution were equivalent between patient groups. The lack of thorough examinations of detailed blood microbiota profiling among participants is another limitation, as we used primers targeting specific species. The primary focus of the present study was on the cbDNA, with no investigation conducted on the compositions of other microorganisms, such as viruses or fungi, which have been recently discovered to be disrupted in fecal samples of CRC patients. Due to these factors, it is advisable to approach the outcomes primarily to generate new hypotheses.

## 4. Materials and Methods

### 4.1. Patients Enrollment and Study Design

From September 2021 to April 2024, 75 patients aged > 18 years old with previously diagnosed and histologically confirmed CRC were included in the present, prospective, multi-centered study. For reference, 25 healthy individuals aged > 18 years old, consisting of pre-graduate medical students from the School of Medicine, University of Crete, who volunteered for this study were also included, without any presence and/or history of benign or malignant neoplastic colorectal disease (Group 1). The patients were equally divided into three groups. Group 2 (*n* = 25) included patients with non-metastatic CRC who underwent surgical resection. Group 3 (*n* = 25) included patients with metastatic CRC who underwent surgical resection of the primary tumor. Group 4 (*n* = 25) included patients with metastatic CRC without surgical resection of the primary tumor.

Further exclusion criteria, which applied to all groups, included the administration of specific medications (antiemetic, antidiarrheal, antiparasitic, antiviral, antibiotic, probiotic, laxatives, non-steroidal anti-inflammatory, or corticosteroid drugs) within 4 weeks prior to sampling, radiographic studies with barium enema within 1 week prior to sampling, active rectal bleeding, individuals with a creatinine clearance (measured by the Cockcroft–Gault equation) < 30 mL/min, pregnancy, presence of any active bacterial or viral infection, synchronous neoplastic disease elsewhere in the body, genetic disease, and CRC due to familial adenomatous polyposis or Lynch syndrome.

All patients were treated at the University Hospital of Heraklion (Department of Medical Oncology, Department of General Surgery, and Department of Surgical Oncology), at the Venizeleio General Hospital of Heraklion (Department of Oncology and Department of Surgery), and at the Creta InterClinic Hospital (Department of Surgery).

### 4.2. Ethics Approval

The study was approved by the Research Ethics Committees of the Creta InterClinic Hospital (9 July 2021, the Venizeleio General Hospital of Heraklion (23/14 July 2023), the University Hospital of Heraklion (23743/2 October 2023), and the University of Crete (119/24 October 2023). Informed consent was provided to and signed by all participants. The conducted procedures adhered to the ethical criteria set by the aforementioned committees.

### 4.3. Blood Sampling and DNA Extraction

Peripheral blood (3 mL in EDTA) was collected from all healthy subjects and CRC patients. Samples were collected just before the initiation of any adjuvant or first-line treatments. NucleoSpin© Blood DNA kit (Macherey-Nagel, Düren, Germany) was used for whole-blood genomic DNA extraction, according to the manufacturer’s protocol. DNA quantification was performed by utilizing the NanoDrop ND-1000 v3.3 (Thermo Fisher Scientific, Waltham, MA, USA) spectrophotometer.

### 4.4. PCR Amplification of Microbial DNA

PCR is the predominant molecular method used to identify pathogens. Typically, for a more complete characterization of the microbiota, NGS is used. However, it should be noted that only a subset of the variable regions is targeted (V1–V3, or V4–V5), which may bias results, leading to the under- or overrepresentation of certain bacterial taxa [60]. It has been stated that PCR-based methods could overcome the amplification bias of NGS, which is crucial in enhancing the reproducibility of the results by targeting genes unique to specific microbial species [61]. Due to the above reasons, PCR was used in our setting as a reliable, highly-sensitive, and cost-effective alternative to NGS for the detection of specific bacterial species of major clinical importance in CRC.

Two oligonucleotide primer pairs were used to detect cbDNA via PCR: the *β*-galactosidase gene of *E. coli* [17,18] and the *NusG* gene of *F. nucleatum*. These primers have also been used in previous reports with conventional PCR-based analysis [17,18,62], yielding high-quality results, thus making them suitable for the purpose of our study. DNA integrity of the samples was performed by using glyceraldehyde phospho-dehydrogenase (GAPDH) as the reference primer [17,18]. For the reference marker in the detection of cbDNA, *16S* rRNA primers were also used in accordance with previous studies [17,18]. The PCR conditions, primer sequences, and fragment size of amplicons for each target gene are reported in Table S1.

### 4.5. Statistical Analysis

Descriptive and inferential statistics were performed regarding the patients’ and healthy individuals’ baseline variables (demographic characteristics and clinical parameters). Comparisons between categorical variables were calculated with Pearson’s chi-square test. Adjusted (adj.) *p*-values through Bonferroni correction were also provided in cases of multiple comparisons. Statistical analysis was performed via SPSS v. 26 (IBM Corp. Armonk, New York, USA). Statistical significance was indicated at the conventional *p* < 0.05 threshold.

## 5. Conclusions

In conclusion, our study confirms the presence of cbDNA in healthy individuals and patients with CRC. High presences of *16S* rRNA and *E. coli* were revealed in all participants, providing strong evidence for bacterial translocation. Moreover, the significantly higher detection of *F. nucleatum* in the blood of patients with metastatic CRC without surgical resection of the primary tumor confirms the role of this bacterium as an intratumoral pathogen associated with advanced stages of CRC. Future studies should focus on correlating these findings with patients’ outcomes and survival, along with additional disease markers (e.g., fecal calprotectin, C-reactive protein [CRP], or cytokines), possibly elucidating the role of *F. nucleatum* as a prognostic biomarker in metastatic CRC. For further investigation of the sources of cbDNA, fecal and oral samples should be analyzed in the future, along with blood microbiota, using more advanced NGS-based methods, assessing the relationship between CRC and microbiota dysbiosis.

## Figures and Tables

**Table 1:** Patients’ and healthy individuals’ demographic characteristics and clinical parameters.

Characteristics	Group 1 (*n* = 25)	Group 2 (*n* = 25)	Group 3 (*n* = 25)	Group 4 (*n* = 25)
	Mean (range)			
Age	20.5 (20–21)	61.5 (25–86)	62.9 (39–80)	57.8 (26–78)
Body Mass Index (BMI) (kg/m^2^)	23.1 (20.3–24.8)	28.4 (22.1–46.9)	27.7 (20.2–37.2)	27.9 (17.1–52.5)
	Frequency (percentage)			
Sex				
Male	18 (72%)	11 (44%)	18 (72%)	16 (64%)
Female	7 (28%)	14 (56%)	7 (28%)	9 (36%)
Surgery				
Yes	n/a *	25 (100%)	25 (100%)	0 (0%)
No	n/a	0 (0%)	0 (0%)	25 (100%)
Metastasis				
Yes	n/a	0 (0%)	25 (100%)	25 (100%)
No	n/a	25 (100%)	0 (0%)	0 (0%)
Tumor Location				
Right Colon	n/a	10 (40%)	10 (40%)	5 (20%)
Left Colon	n/a	9 (36%)	8 (32%)	10 (40%)
Rectum	n/a	6 (24%)	7 (28%)	10 (40%)
Stage				
II	n/a	5 (20%)	0 (0%)	0 (0%)
III	n/a	20 (80%)	0 (0%)	0 (0%)
IV	n/a	0 (0%)	25 (100%)	25 (100%)
Grade				
Low	n/a	6 (24%)	2 (8%)	2 (8%)
Intermediate	n/a	13 (52%)	15 (60%)	16 (64%)
High	n/a	6 (24%)	8 (32%)	7 (28%)
Mismatch Repair (MMR) status				
Microsatellite Stability (MSS)	n/a	19 (76%)	23 (92%)	21 (84%)
Microsatellite Instability (MSI)	n/a	3 (12%)	2 (8%)	2 (8%)
Unreported	n/a	3 (12%)	0 (0%)	2 (8%)
Smoking				
Yes	0 (0%)	7 (28%)	12 (48%)	8 (32%)
No	25 (100%)	18 (72%)	13 (52%)	17 (68%)
Alcohol				
Yes	0 (0%)	1 (4%)	3 (12%)	4 (16%)
No	25 (100%)	24 (96%)	22 (88%)	21 (84%)
Diabetes				
Yes	0 (0%)	5 (20%)	11 (44%)	3 (12%)
No	25 (100%)	20 (80%)	14 (56%)	22 (88%)
Dyslipidemia				
Yes	0 (0%)	9 (36%)	5 (20%)	6 (24%)
No	25 (100%)	16 (64%)	20 (80%)	19 (76%)
Cardiovascular Disease (CVD)				
Yes	0 (0%)	12 (48%)	13 (52%)	9 (36%)
No	25 (100%)	13 (52%)	12 (48%)	16 (64%)

* n/a, not applicable.

**Table 2:** Frequencies and percentages of circulating bacterial DNA (cbDNA) presence among control and patient groups.

Gene Target	Detection	Group 1 (*n* = 25)	Group 2 (*n* = 25)	Group 3 (*n* = 25)	Group 4 (*n* = 25)
*16S* rRNA	Positive	21 (84%)	24 (96%)	25 (100%)	25 (100%)
	Negative	4 (16%)	1 (4%)	0 (0%)	0 (0%)
*β*-galactosidase gene of *Escherichia coli*	Positive	20 (80%)	20 (80%)	21 (84%)	17 (68%)
	Negative	5 (20%)	5 (20%)	4 (16%)	8 (32%)
*NusG* gene of *Fusobacterium nucleatum*	Positive	3 (12%)	8 (32%)	8 (32%)	17 (68%)
	Negative	22 (88%)	17 (68%)	17 (68%)	8 (32%)

**Table 3:** Comparisons of cbDNA detection among control and patient groups.

Pairwise Comparison	*16S* rRNA		*E. coli*		*F. nucleatum*	
	*p*-Value	adj. *p*-Value	*p*-Value	adj. *p*-Value	*p*-Value	adj. *p*-Value
Group 1–Group 2	0.349	0.697	- *	-	0.171	0.205
Group 1–Group 3	0.11	0.33	-	-	0.171	0.205
Group 1–Group 4	0.11	0.33	0.52	-	<0.001	<0.001
Group 2–Group 3	-	-	-	-	-	-
Group 2–Group 4	-	-	0.52	-	0.023	0.045
Group 3–Group 4	-	-	0.321	-	0.023	0.045

* *p* = 1.

**Table 4:** Association of cbDNA detection with patients’ clinical parameters.

Parameter/Pairwise Comparison	*E. coli*		*F. nucleatum*	
	*p*-Value	adj. *p*-Value	*p*-Value	adj. *p*-Value
Sex				
Female–Male	0.403	n/a ^1^	0.348	n/a
Surgery				
No–Yes	0.242	n/a	0.006	n/a
Metastasis				
No (stage II/III)–Yes (stage IV)	0.777	n/a	0.217	n/a
Tumor Location				
Left Colon–Rectum	0.308	0.462	0.252	0.596
Right Colon–Rectum	0.292	0.462	- ^2^	-
Left Colon–Right Colon	-	-	0.397	0.596
Grade				
High–Low	0.634	0.951	0.701	-
Intermediate–Low	0.427	0.951	-	-
High–Intermediate	-	-	0.605	-
MMR status				
MSI–MSS	-	n/a	-	n/a
Smoking				
No–Yes	0.15	n/a	0.634	n/a
Alcohol				
No–Yes	-	n/a	0.725	n/a
Diabetes				
No–Yes	0.004	n/a	0.286	n/a
Dyslipidemia				
No–Yes	-	n/a	-	n/a
CVD				
No–Yes	0.414	n/a	0.816	n/a

^1^ n/a, not applicable; ^2^ *p* = 1.

## Data Availability

All data used for the analysis of the current study are provided in Supplementary Data S1.

## Supplementary Materials

The following supporting information can be downloaded at: https://www.mdpi.com/article/10.3390/ijms25169025/s1.
