# Supplementary material for: Circulating Bacterial DNA in Colorectal Cancer Patients: The Potential Role of Fusobacterium nucleatum
Source: Int J Mol Sci. 2024 Aug 20;25(16):9025. doi: 10.3390/ijms25169025 (PMC11354820; doi:10.3390/ijms25169025)
Supplement: Supplementary file 1 [file ijms-25-09025-s001.zip › Figure S1.pdf]

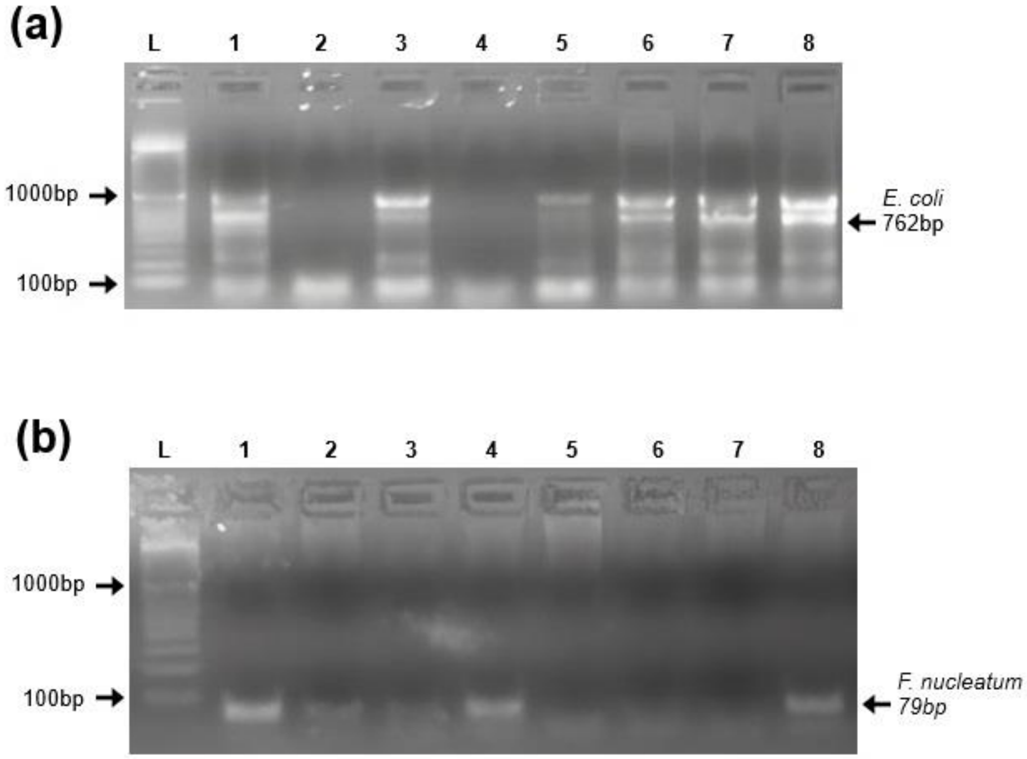

Figure S1. Agarose gel electrophoresis examples of PCR products regarding the detection of circulating bacterial DNA in blood. (a) Detection of *β*-galactosidase gene of *Escherichia coli* (*E. coli*): positive (lanes 1,3,5-8); negative (lanes 2,4). (b) Detection of *NusG* gene of *Fusobacterium nucleatum* (*F. nucleatum*): positive (lanes 1-4,8); negative (lanes 5-7). L: 100bp ladder.
