# Supplementary material for: Circulating Bacterial DNA in Colorectal Cancer Patients: The Potential Role of Fusobacterium nucleatum
Source: Int J Mol Sci. 2024 Aug 20;25(16):9025. doi: 10.3390/ijms25169025 (PMC11354820; doi:10.3390/ijms25169025)
Supplement: Supplementary file 1 [file ijms-25-09025-s001.zip › Table S1.pdf]

Supplementary Table S1. PCR primers and conditions for DNA amplification

| Target Gene | Primer | Sequence | PCR Conditions | Fragment Size |
|---|---|---|---|---|
| GAPDH | F | 5'-TCTCCAGAACATCATCCTG-3' | Denaturation at 95 °C for 5 min. Then, samples were exposed to 35 cycles of denaturing (95 °C, 1 min), annealing (60 °C, 1 min), and extension (72 °C, 1 min), followed by a final extension step at 72 °C for 10 min | 324 bp |
| | R | 3'-TGCTGGTGAAACAGTTCGAG-5' | | |
| *16S* ribosomal RNA for Gram+ and Gram- bacteria | F | 5'-AGTTTGATCCTGGCTCAG-3' | | 798 bp |
| | R | 3'-TAATCTATGGGACCATCAGG-5' | | |
| *β*-galactosidase gene of *Escherichia coli* | F | 5'-CTTGCCTGGTTTCCGGCACCAGAA-3' | | 762 bp |
| | R | 3'-GGGCTTAGAGATAGCACGCCACCAA-5' | | |
| *NusG* gene of *Fusobacterium nucleatum* | F | 5'-CAACCATTACTTTAACTCTACCATGTTCA-3' | | 79 bp |
| | R | 3'-GTTGACTTTACAGAAGGAGATTATGTAAAAATC-5' | | |
